# Template-Free Electrochemical Growth of Ni-Decorated ZnO Nanorod Array: Application to an Anode of Lithium Ion Battery

**DOI:** 10.3389/fchem.2019.00415

**Published:** 2019-06-06

**Authors:** Han Nah Park, Sun Hwa Park, Jeong Ho Shin, Soo-Hwan Jeong, Jae Yong Song

**Affiliations:** ^1^Center for Convergence Property Measurement, Korea Research Institute of Standards and Science, Daejeon, South Korea; ^2^Department of Chemical Engineering, Kyungpook National University, Daegu, South Korea

**Keywords:** ZnO, nanorod, electrochemical property, spherical PVDF, Ni nanoparticle

## Abstract

ZnO nanorods (NRs) decorated with Ni nanoparticles were synthesized using a template-free electrochemical deposition in an ultra-dilute aqueous electrolyte and a subsequent galvanic reaction. The electrochemical properties of the ZnO NRs as an anode material for rechargeable Li-ion batteries were evaluated for different binder morphologies (film and close-packed spherical particles) of polyvinylidene fluoride (PVDF). Results showed that the close-packed spherical PVDF simultaneously improved electrochemical capacity and cyclability because the free-volume between the spherical PVDF helped to accommodate the volume change in the anode caused by the Li ions discharge and charge processes. Furthermore, the Ni nanoparticles decorated on the surface of ZnO NRs enhanced the electrical conductivity of the ZnO NR anode, which enabled faster electronic and ionic transport at the interface between the electrolyte and the electrode, resulting in improved electrochemical capacity. The free-volume formed by the close-packed spherical PVDF, and the decoration of metal nanoparticles are expected to provide insight on the simultaneous improvement of electrochemical capacity and cyclability in other metal oxide anode nanostructures.

## Introduction

Rechargeable Li-ion batteries (LIBs) have been widely used for energy storage in portable devices such as cellular phones, cameras, and lap-top computers (Tarascon and Armand, 2001; Li et al., 2009; Scrosati and Garche, 2010). However, there are still several challenging issues that need to be resolved to improve LiB performance. For example, to meet the increasing demand for LiBs with higher energy density, the capacity of the anode must be improved, because the Li storage capacity of commercialized graphite anode is limited to the theoretical maximum capacity of 372 mAh/g (Tokumitsu et al., 1999; Li et al., 2009; Liu and Cao, 2010). For this reason, extensive studies have focused on developing new anode materials to replace graphite. Transition metal oxides (MO, M = Fe, Co, Ni, Cu, and Zn) in particular have attracted much attention because the electrochemical capacities of these materials are two times higher than that of graphite (Park et al., 2009; Huang et al., 2012; Caballero et al., 2013; He et al., 2013; Yanga et al., 2013). Among them, ZnO has several advantages, including high theoretical capacity (978 mAh/g), low cost and chemical stability (Zhang et al., 2007, 2016; Pan et al., 2010; Huang et al., 2011). Unfortunately, ZnO has a critical drawback. The anode is mechanically disintegrated by the large volume expansion and shrinkage that occurs with the reaction of ZnO and Li, and this degrades structural stability and electrical conductivity, affecting electrochemical performance (Belliard and Irvine, 2001; Laurenti et al., 2015; Song et al., 2017). To avoid this degradation, several nanostructures have been investigated in anodes, including nanorods, nanosheets, nanoparticles, and porous nanostructures (Belliard and Irvine, 2001; Zhang et al., 2007; Huang et al., 2011, 2014a; Laurenti et al., 2015). Vertically aligned nanorods are expected to accommodate the huge volume changes, while providing faster transport of charge carriers, due to the free volume between the nanorods, high specific surface area and short diffusion path (Liu et al., 2009; Wang et al., 2009).

In the present study, we were motivated to synthesize a vertically aligned ZnO NR array using a low-cost template-free electrochemical deposition method, which was developed to grow a nanorod array of pure metals (Au, Ag, and Cu) and metal oxide (Cu_2_O) (Park et al., 2013a,b; Shin et al., 2014). And, instead of using a polyvinylidene fluoride (PVDF) film structure, close-packed spherical nanoparticles of PVDF (sPVDF) were used to reduce the large volume expansion caused by Li intercalation. For faster transport of charge carriers, Ni nanoparticles were decorated on the surface of ZnO NRs using electroless deposition. The improved electrochemical performance of the sPVDF-embedded ZnO NRs decorated with Ni nanoparticles was then investigated.

## Experimental

### Preparation of ZnO Nanostructures

ZnO NRs were synthesized using a potentiodynamic electrodeposition process. The electrodeposition was carried out with a three-electrode system (Solartron 1280z). The working electrode was Cu foil (99.8 at%, Nippon Foil Mfg, Co.) with an exposed area of 1.5 × 1.5 cm^2^. Pt wire (Aldrich) and KCl–saturated Ag/AgCl were used as the counter and reference electrodes, respectively. The electrolyte was composed of 100 μM ZnCl_2_ (Aldrich) in deionized water and set at 40°C. The pH and conductivity of the electrolyte were 5.5 and 24 μS/cm, respectively. In the potentiodynamic mode, the reduction potential (V_R_) and oxidation potential (V_O_) were set to be −8 V and +0.5 V. The duty and frequency of the potentiodynamic mode were 50% and 1 Hz, respectively. For the deposition of Ni nanoparticles on the surface of ZnO NRs, we conducted electroless Ni plating in an aqueous solution of 95 mM NiSO_4_·6H_2_O and 284 mM NaPH_2_O_2_. The pH value of the solution was set to be 6.0 by adding 21.9 mM NaOH. Commercial sPVDF (Kynar HSV 900) nanoparticles were used. The sPVDF nanoparticles were dispersed in 15 wt% acetone and several spin-coatings were conducted until they were completely infiltrated between the NRs.

### Structural Characterization

A field emission scanning electron microscope (FE–SEM, Hitachi S−4800) was employed for the morphological characterization of the samples. The crystal structures and components of the ZnO nanostructures were analyzed using x-ray diffraction (XRD, Cu–K, Rigaku D/max–B), field emission transmission electron microscope (FE–TEM, FEI Tecnai F30), and energy dispersive x-ray spectrometry (EDS, EDAX Genesis XM4).

### Measurement of Electrochemical Properties

The electrochemical properties of the ZnO nanostructures as an LIB anode were investigated using a CR2032 coin cell. Li metal foil was used as a counter electrode and the separator was microporous polyethylene (Celgard 2400). The electrolyte was a 1 mol/L LiPF_6_ in a 1:1 (v/v ratio) mixture of ethylene carbonate and diethyl carbonate (Techno Semichem Co.). Galvanostatic charge-discharge tests were carried out using an automatic battery test system (Wonatech Co., WBCS3000S) in the voltage window of 0.05–2.4 V (vs. Li/Li^+^) with a rate of 0.5 C. The cyclic voltammetry (CV) measurement was conducted at a rate of 1 mV/s. The electrochemical impedance spectrometry (EIS) measurement was carried out with a range of 0.1 Hz to 100 kHz.

## Results and Discussions

**Figure 1:**
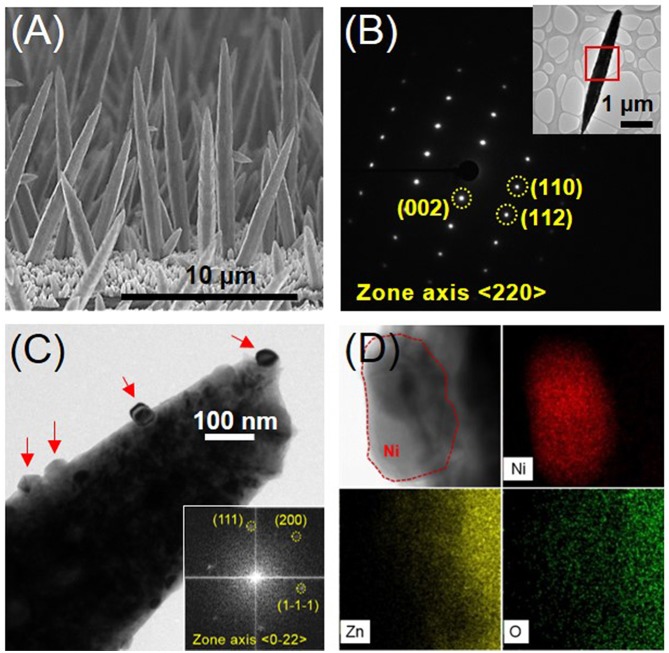
**(A)** Cross-sectional SEM image of the ZnO NRs grown on Cu foil, **(B)** SAED (Selected Area Electron Diffraction) pattern of the position marked in the inset of a single ZnO nanorod, **(C)** BF (bright field) TEM image of Ni-decorated ZnO NR (The right inset indicates the FFT (Fast Fourier Transform) pattern of a Ni nanoparticle, corresponding to the red-dashed position in the left inset TEM image, on the surface of a ZnO NR.) **(D)** BFTEM image and EDS mappings according to the compositional elements Ni, Zn, and O on the surface of Ni-coated ZnO NRs.

Figure 1 shows the morphological and crystal structure characterization of the ZnO NRs and the Ni nanoparticle-decorated ZnO NRs. Short ZnO NRs (less than 500 nm in length and 200 nm in diameter) were densely grown on Cu substrates, and then long ZnO NRs (more than 10 μm in length and 800 nm in diameter) were sparsely grown, as shown in Figure 1A. Sharp ZnO NRs were grown, with an apex on top (Qiu et al., 2011; Spitsina and Kahrizi, 2012). This might suggest that the growth of the ZnO NRs was caused by electric field enhancement in the dilute electrolyte (Elias et al., 2008). The possible reactions during the electrodeposition of ZnO NRs in the acidic electrolyte can be described, as follows (Qiu et al., 2011; Spitsina and Kahrizi, 2012):

(1) $$\begin{array}{r} O_{2}+2H_{2}O+4e^{-}\to 4OH^{-} \end{array}$$

(2) $$\begin{array}{r} Zn^{2+}+2OH^{-}\to Zn{\left(OH\right)}_{2} \end{array}$$

(3) $$\begin{array}{r} Zn{\left(OH\right)}_{2}\to ZnO+H_{2}O \end{array}$$

According to Equation (1), water decomposition generates hydroxyl ions (OH^−^) near the cathode, where an alkaline state is locally formed (Manzano et al., 2013). Zn cations in the electrolyte react with hydroxyl ions, and zinc hydroxide is formed following Equation (2). Subsequently, the zinc hydroxide undergoes a dehydration process, as shown in Equation (3). This might be ascribed to the high electric field in the dilute electrolyte, which is similar to the previous result, which found that Cu_2_O nanorods grew in a dilute acidic electrolyte of copper sulfate (Shin et al., 2014).

The ZnO NRs had a hexagonal crystal structure (JCPDS # 36-1451) and grew in the longitudinal <002> direction, as indexed in the TEM analyses shown in Figure 1B. The preferred growth of the ZnO NRs was confirmed by XRD analyses, which indicated that the (002) peak was the strongest (see supporting information, Figure S1). Figure 1C shows that Ni nanoparticles (20–50 nm in size) marked by red arrows have formed on the surface of the ZnO NRs, obtained from the EDS mapping analyses in Figure 1D. XRD peaks were not observed for the Ni phase due to the small amount of Ni nanoparticles (Figure S1). However, the TEM analyses of the Ni nanoparticle shown in the inset of Figure 1C was indexed to have a face-centered cubic crystal structure (JCPDS # 04-0850) and the Ni-decorated ZnO NRs had identical crystal structures. The electroless deposition of Ni nanoparticles by hypophosphite reduction in nickel sulfate is represented as follows (Iniewski, 2011).

(4) $$\begin{array}{r} Ni^{2+}+H_{2}PO_{2^{-}}+H_{2}O\to Ni+2H^{+}+H{\left(HPO_{3}\right)}^{-} \end{array}$$

**Figure 2:**
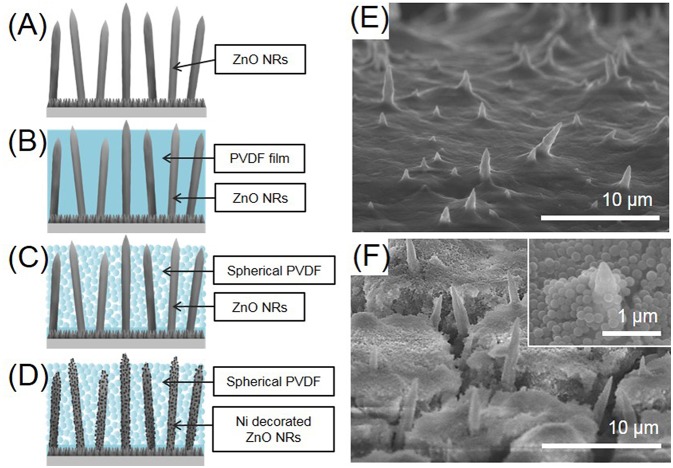
Schematic diagrams of **(A)** as-prepared ZnO NRs, **(B)** ZnO NRs embedded in PVDF film, **(C)** sPVDF-infiltrated ZnO NRs, and **(D)** sPVDF-infiltrated ZnO NRs decorated with Ni nanoparticles. The top-view SEM image of **(E)** ZnO NRs in PVDF film and **(F)** sPVDF-infiltrated ZnO NRs.

The ZnO NRs directly grown on Cu foil were then electrochemically evaluated as an anode in an LIB. Four kinds of anodes were prepared, for comparison, i.e., as-prepared ZnO NRs, ZnO NRs embedded in PVDF film, sPVDF-infiltrated ZnO NRs, and sPVDF-infiltrated ZnO NRs decorated with Ni nanoparticles, respectively, as shown in Figures 2A–D. The typical top-view SEM images of ZnO NRs in PVDF film and ZnO NRs in sPVDF particles are shown in Figures 2E,F, respectively. The ZnO NRs were fully embedded in the PVDF film, as shown in Figure 2E, while the sPVDF particles were densely packed between ZnO NRs by several spin-coatings, as shown in Figure 2F. As shown in the inset of Figure 2F, the ZnO NRs were surrounded by the sPVDF particles with an average size of 150 nm.

**Figure 3:**
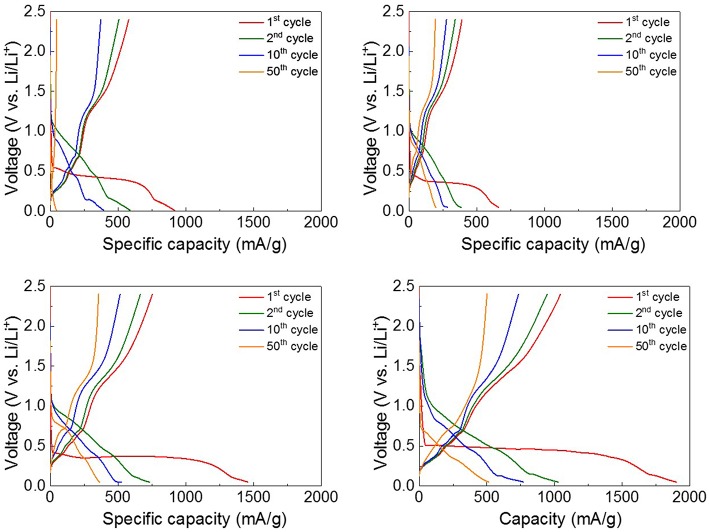
The discharge-charge voltage profiles between 0.05 and 2.4 V of **(A)** as-prepared ZnO NRs, **(B)** ZnO NRs embedded in PVDF film, **(C)** sPVDF-infiltrated ZnO NRs, and **(D)** sPVDF-infiltrated ZnO NRs decorated with Ni nanoparticles.

Figure 3 shows the discharge-charge voltage profiles of the four ZnO NRs samples obtained between 0.05 and 2.4 V (vs. Li/Li^+^) at the rate of 0.5 C. According to previous studies (Zhang et al., 2007; Huang et al., 2011; Laurenti et al., 2015), the electrochemical Li-storage mechanism of ZnO is composed of two reversible reactions, as noted below.

(5) $$\begin{array}{r} ZnO+2Li^{+}+2e^{-}↔Zn+Li_{2}O \end{array}$$

(6) $$\begin{array}{r} Zn+xLi^{+}+xe^{-}↔Li_{\text{x}}Zn\;\left(x\leq 1\right) \end{array}$$

The behavior of the voltage profiles of the four ZnO NRs anodes was similar. The 1st discharge curves of the four anodes exhibited long voltage plateaus at 0.5 V, but the 1st charge curves showed shorter plateaus at 1.4 V. It can be seen that the 1st voltage profiles are somewhat irreversible due to the typical characteristics of transition-metal oxides (Huang et al., 2014b). In particular, the sPVDF-infiltrated ZnO NRs and the sPVDF-infiltrated ZnO NRs decorated with Ni nanoparticles exhibited 1st discharge capacities of 1,459 and 1,902 mAh/g, which are higher than the theoretical value of ZnO (978 mAh/g). The excess capacities are caused by the formation of irreversible Li_2_O and a solution electrolyte interface (SEI) layer, as well as electrolyte decomposition in the low potential window (Zhang et al., 2007; Huang et al., 2014b; Shen and Wang, 2015). After the 2nd voltage profiles, the reactions of the four anodes became more reversible. And the plateaus on the voltage profiles coincided with the peaks in the CV curves.

Figure S2A shows the CV curves of sPVDF-infiltrated ZnO NRs between 0.05 and 2.4 V (vs. Li/Li^+^) at a scan rate of 1 mV/s. In the 1st cathodic reaction, there is only one peak below 0.3 V due to the irreversible reaction. For the 2nd and 5th cycles, the CV curves indicate a more reversible electrode reaction since the curves almost coincide in shape. The CV curves also exhibit typical reduction and oxidation peaks, indicating the Li-storage mechanism of ZnO. In the cathodic curve, the reduction peaks in the potential range of 0–0.7 V correspond to the formation of Li-Zn alloy and a solid electrolyte interphase (SEI) layer as well as the reduction of ZnO to Zn, yielding Li_2_O. In the anodic curve, the oxidation peaks in the potential range below 0.8 V are related to the multi-step dealloying of the Li-Zn alloy. And the strong broad oxidation peak located near 1.5 V is associated with the decomposition of Li_2_O (Zhang et al., 2007; Ahmad et al., 2011; Huang et al., 2014a; Xie et al., 2015). The CV curve of the sPVDF-infiltrated ZnO NRs is quite similar to those of the as-prepared ZnO NRs and ZnO NRs embedded in PVDF film as shown in Figure S2B. And the CV curve for the sPVDF-infiltrated ZnO NRs decorated with Ni nanoparticles showed a higher current intensity near 0.5 V of the cathodic peak and 1.5 V of the anodic peak. It may be possible to improve the oxidation-reduction reaction of the Li-Zn alloy as well as the reduction of Li_2_O, by using Ni as a catalyst, affecting the capacity (Zhang et al., 2007; Ahmad et al., 2011; Huang et al., 2014a;Xie et al., 2015).

**Figure 4:**
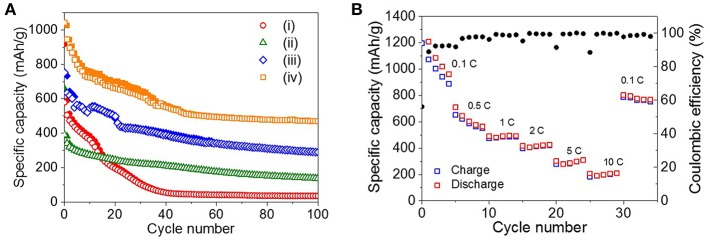
**(A)** Cycling performance of (i) as-prepared ZnO NRs, (ii) ZnO NRs embedded in PVDF film, (iii) sPVDF-infiltrated ZnO NRs, and (iv) sPVDF-infiltrated ZnO NRs decorated with Ni nanoparticles over 100 cycles at 0.5 C. (The solid and open symbols indicate charge and discharge capacities, respectively). **(B)** Rate capability of (iv) sPVDF-infiltrated ZnO NRs decorated with Ni nanoparticles.

The long-term cycling performance of the four ZnO NRs samples was measured at the rate of 0.5 C for 100 cycles, as shown in Figure 4Ai. For the as-prepared ZnO NRs, the specific capacity drastically decreased after 40 cycles, and the poor discharge capacity retention of 36 mAh/g was observed after 100 cycles.

Since the reactions are accompanied by the huge theoretical volume change of ~228%, the volume change-driven mechanical stress tends to deteriorate the anode (Figure S3A) (Xie et al., 2015). To suppress the disintegration of the ZnO NRs, the PVDF film was used to fill the gap between the ZnO NRs as a binder. In Figure 4Aii, the ZnO NRs embedded in the PVDF film show more stable cycling behavior as a result. However, the maximum discharge capacity value of the ZnO NRs embedded in the PVDF film was about 44% lower than that of the as-prepared ZnO NRs. It is noted that the PVDF, which completely surrounds the ZnO NRs, suppresses the volume expansion of the ZnO NRs caused by lithiation process, and interferes with the diffusion of Li ions between the ZnO NRs and electrolyte during the charge/discharge processes. Figure S3B and Figure 2E show that the PVDF film with embedded ZnO NRs retained its initial shape, even after 100 cycles.

As shown in Figure 4Aiii, spherical nanoparticles of PVDF (sPVDF) were infiltrated between the ZnO NRs to facilitate the diffusion of Li ions and to allow greater volume expansion, i.e., more electrochemical capacity caused by the lithiation process. Figure 4Aiii shows that the sPVDF-infiltrated ZnO NRs had an average specific capacity of around 406 mAh/g, which is two times higher than that of the ZnO NRs embedded in the PVDF film.

In addition, the 2nd discharge capacity of the sPVDF-infiltrated ZnO NRs was 143 mAh/g higher than that (about 589 mAh/g) of the as-prepared ZnO NRs. This result might be explained if the free-volume formed by the sPVDF contributed to the fast intercalation and de-intercalation of Li^+^ ions between the electrolyte and the ZnO NRs (Gowda et al., 2011). After 100 cycles, the specific capacity of the sPVDF-infiltrated ZnO NRs was two times higher than that of the ZnO NRs embedded in the PVDF film and eight times higher than that of the as-prepared ZnO NRs. It is supposed that the close-packed sPVDF acted as a binder and effectively prevented the delamination of the ZnO NRs from the Cu foil as shown in Figure S3C. Figures S3D,E show SEM images of the interface between the ZnO NRs and Cu foil before and after the 100 cycles of the sPVDF-infiltrated ZnO NRs. This indicates that the ZnO NRs were well-preserved in the initial structure without delamination after the 100 cycling charge-discharge process. This confirms that the close-packed sPVDF-infiltrated ZnO NRs are an effective structure for accommodating the strains caused by the electrochemical processes, and simultaneously allow enhanced electrochemical performances.

Figure 4Aiv shows the specific capacity of the sPVDF-infiltrated ZnO NRs decorated with Ni nanoparticles. The average specific capacity for 100 cycles was about 595 mAh/g, 1.5 times higher than that of the sPVDF-infiltrated ZnO NRs. And the discharge capacity at the 2nd cycle increased more than 20 %, compared to that of the sPVDF-infiltrated ZnO NRs. This indicates that the decoration of Ni nanoparticles improved the electrochemical performance of the ZnO NRs (Zhang et al., 2007; Xie et al., 2015).

Figure 4B shows the rate capability of the sPVDF-infiltrated ZnO NRs decorated with Ni nanoparticles. The average discharge capacities at the rates of 0.1, 0.5, 1, 2, 5, and 10 C are 1,280, 619, 490, 417, 295, and 202 mAh/g, respectively. The capacity gradually decreases at 0.1 and 0.5 C. However, when the c-rate returns back to 0.1 C from 10 C, this anode showed a recovered capacity up to 782 mAh/g and the high coulomb efficiency of 98%, indicating the good stability. At high rates of 1, 2, 5, and 10 C, this anode has high capacity compared to the previous studies, and good cycle characteristics for the fast discharge-charge feature (Pan et al., 2010; Ahmad et al., 2011; Gowda et al., 2011; Laurenti et al., 2015).

**Figure 5:**
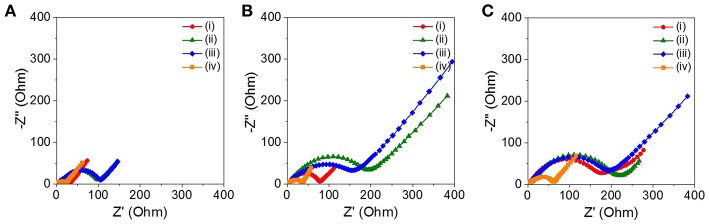
Nyquist plots of the cell with the (i) as-prepared ZnO NRs, (ii) ZnO NRs embedded in PVDF film, (iii) sPVDF-infiltrated ZnO NRs, and (iv) sPVDF-infiltrated ZnO NRs decorated with Ni nanoparticles at the end of the **(A)** 10th, **(B)** 30th, and **(C)** 50th charge cycles.

Figure 5 shows the Nyquist plots of the four ZnO NRs at the end of the 10, 30, and 50th charge cycles, respectively. The solution resistance (R_s_) and the charge transfer resistance (R_ct_) were estimated by a semicircle in the high-to-medium region and by a simple equivalent circuit model. The four ZnO NRs samples had similar R_s_ values, in the range of 2–4 Ω, regardless of the charge cycles. The sPVDF-infiltrated ZnO NRs decorated with Ni nanoparticles and the as-prepared ZnO NRs exhibited an R_ct_ value (22 and 33 Ω) at the 10th charge cycle, which was much smaller than that those (105 and 100 Ω) of the sPVDF-infiltrated ZnO NRs, and the ZnO NRs embedded in PVDF film. With the increase of the charge cycles, the R_ct_ values of the sPVDF-infiltrated ZnO NRs and the ZnO NRs embedded in PVDF film increased to 178 and 205 Ω in the 30th cycle, reaching 221 and 234 Ω in the 50th cycle, respectively. And the R_ct_ value of the as-prepared ZnO NRs slightly increased to 79 Ω in the 30th cycle and reached 200 Ω in the 50th cycle, similar to the R_ct_ values of the sPVDF-infiltrated ZnO NRs and the ZnO NRs embedded in PVDF film. Even though the R_ct_ values of the sPVDF-infiltrated ZnO NRs decorated with Ni nanoparticles slightly increased with the charge cycling, it had a much lower R_ct_ than the others. This suggests that the presence of Ni nanoparticles increased the electrical conductivity of the ZnO NRs as an active material, and facilitated a faster kinetics process toward the formation/decomposition of Li_2_O, thus leading to high specific capacity (Zhang et al., 2007; Liu et al., 2009;Huang et al., 2014b).

## Conclusions

In summary, we successfully synthesized ZnO NRs decorated with Ni nanoparticles using a template-free electrochemical deposition and subsequent galvanic reaction. The specific electrochemical capacities of the four specimens, i.e., as-prepared ZnO NRs, ZnO NRs embedded in PVDF film, sPVDF-infiltrated ZnO NRs, and sPVDF-infiltrated ZnO NRs decorated with Ni nanoparticles, were evaluated as anodes for LIB, respectively. The close-packed sPVDF infiltrated between the ZnO NRs, as well as the Ni nanoparticles decorated on the ZnO NR surface, led to the simultaneous enhancement of electrochemical capacity and cyclability as an anode material. The free-volume formed by the sPVDF contributed to accommodation of the strain induced by the fast intercalation/de-intercalation of Li^+^ ions. And the Ni nanoparticles deposited on the surface of the ZnO NRs both increased electrical conductivity, and facilitated a faster kinetics in the process of the formation/decomposition of Li_2_O. The present results are expected to contribute to the enhancement of the electrochemical performance of other metal oxide nanostructures.

## Author Contributions

HP and SP conducted the experiments and TEM analysis and drafted the manuscript. JHS analyzed the electrochemical properties. S-HJ and JS initiated and organized the work and finalized the manuscript. All authors read and approved the final manuscript.

### Conflict of Interest Statement

The authors declare that the research was conducted in the absence of any commercial or financial relationships that could be construed as a potential conflict of interest.
