# Supplementary material for: Template-Free Electrochemical Growth of Ni-Decorated ZnO Nanorod Array: Application to an Anode of Lithium Ion Battery
Source: Front Chem. 2019 Jun 6;7:415. doi: 10.3389/fchem.2019.00415 (PMC6563756; doi:10.3389/fchem.2019.00415)
Supplement: Supplementary file 1 [file Data_Sheet_1.docx]

Supplementary Material

Template-free Electrochemical Growth of Ni-decorated ZnO Nanorod Array: Application to an Anode of Lithium Ion Battery

Han Nah Park ^1,2§^, Sun Hwa Park ^1§^, Jeong Ho Shin ^1^, Soo-Hwan Jeong ^2,*^, Jae Yong Song ^1,*^

^1^Center for Convergence Property Measurement, Korea Research Institute of Standards and Science, Daejeon, Republic of Korea

^2^Department of Chemical Engineering, Kyungpook National University, Daegu, Republic of Korea

**^§^ Equal contribution**

*** Correspondence:**E-mail : jysong@kriss.re.kr (Jae Yong Song), shjeong@knu.ac.kr (Soo-Hwan Jeong)

**Supplementary data**

**Figure S1.** XRD spectra of ZnO NRs and (block) and Ni-decorated ZnO NRs (gray) grown on the Cu foil. (Si comes from the supporting substrate)

**Figure S2.** CV curves of **(A)** the 1^st^, 2^nd^ and 5^th^ cycles of the sPVDF-infiltrated ZnO NRs and **(B)** the 5^th^ cycles for the (i) as-prepared ZnO NRs, (ii) ZnO NRs embedded in PVDF film, (iii) sPVDF-infiltrated ZnO NRs and (iv) sPVDF-infiltrated ZnO NRs decorated with Ni nanoparticles at a scan rate of 1 mV/s.

**Figure S3.** SEM images of **(A)** as-prepared ZnO NRs, **(B)** ZnO NRs embedded in PVDF film, and **(C)** sPVDF-infiltrated ZnO NRs after 100 cycles at the rate of 0.5 C. The magnified SEM images of spherical PVDF particles and buffer layers before **(D)** and after **(E)** the 100 cycles of sPVDF-infiltrated ZnO NRs.
