# Supplementary figures and images for: Template-Free Electrochemical Growth of Ni-Decorated ZnO Nanorod Array: Application to an Anode of Lithium Ion Battery
Source: Front Chem. 2019 Jun 6;7:415. doi: 10.3389/fchem.2019.00415 (PMC6563756; doi:10.3389/fchem.2019.00415)

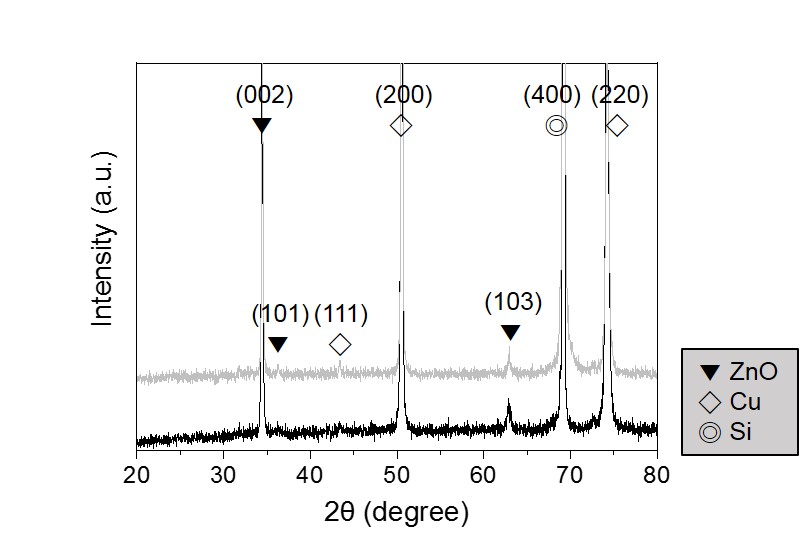

Supplement: Supplementary file 2 [file Image_1.JPEG]

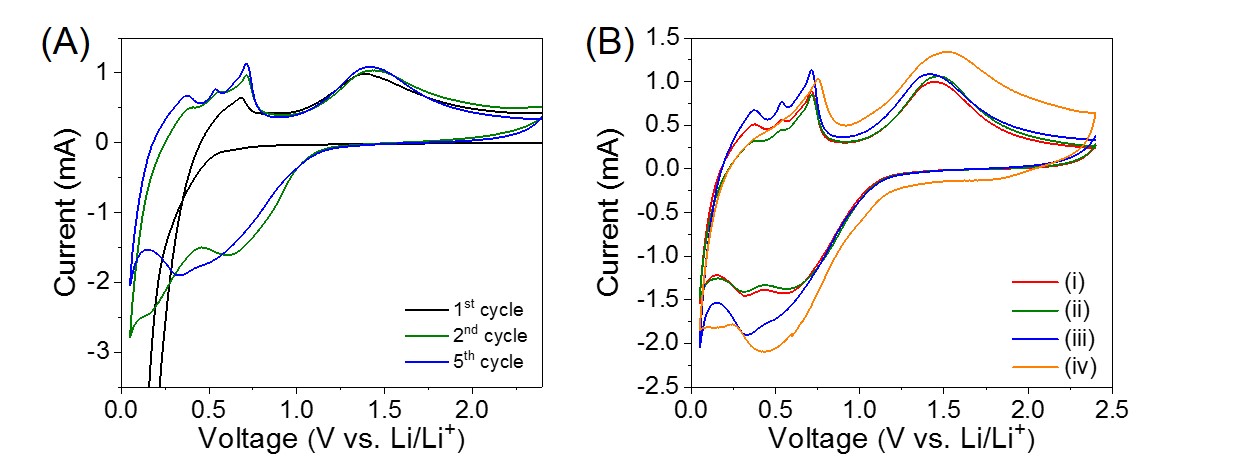

Supplement: Supplementary file 3 [file Image_2.JPEG]

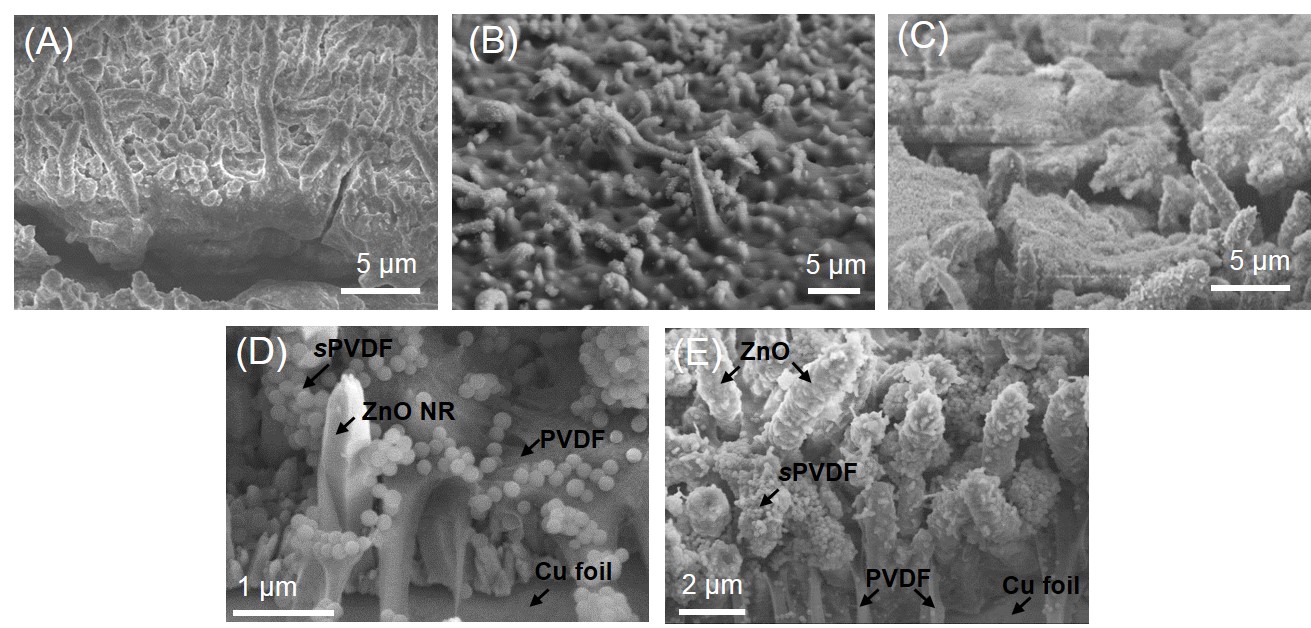

Supplement: Supplementary file 4 [file Image_3.JPEG]
